# Kalzifizierender fibröser Tumor des Thorax: Seltener Tumor mit typischer Histomorphologie

**DOI:** 10.1007/s00292-025-01469-z

**Published:** 2025-09-30

**Authors:** Tereza Losmanova, Sabina Berezowska

**Affiliations:** 1 Institut für Gewebemedizin und Pathologie, Universität Bern, Bern, Schweiz; 2 Department of Laboratory Medicine and Pathology, Institute of Pathology, Lausanne University Hospital and University of Lausanne, Lausanne, Schweiz

​

**Abb. 1:**
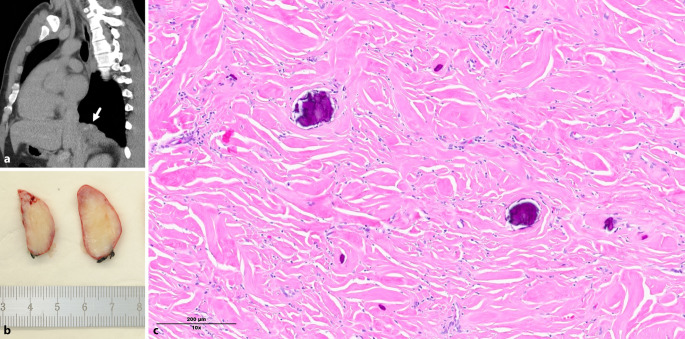
Beim 23-jährigen Mann zeigten sich im Rahmen der diagnostischen Abklärung nach einem Trauma in der CT-Untersuchung multiple pleurale Knoten, wobei der größte Knoten mit 6,4 × 2,7 × 4,4 cm am rechten Unterlappen mit Ausdehnung zum Mediastinum lag (in der Abbildung **a** durch einen *Pfeil* markiert). Klinisch wurden ein Paragangliom, ein Neurofibrom oder eine kongenitale pulmonale Atemwegsmalformation (CPAM) diskutiert. Nach erfolgloser endoskopischer Feinnadelaspiration wurde eine thorakoskopische Operation mit Resektion des größten Knotens durchgeführt. Makroskopisch zeigte sich ein scharf begrenzter Tumor mit homogener, weißer Schnittfläche (**b**). Histologisch bestand die Läsion aus dichter, keloidähnlicher Fibrose mit multiplen Verkalkungen und fokalen lymphoplasmazellulären Infiltraten (**c**). Die immunhistochemische Untersuchung war nicht wegweisend, insbesondere wurden keine IgG4-positiven Infiltrate identifiziert. Die Kongorot-Färbung fiel negativ aus. Insgesamt entsprach das Bild einem kalzifizierenden fibrösen Tumor des Thorax. Diese seltene, gutartige Neoplasie, die typischerweise als pleuraständige Raumforderung auftritt, wurde erstmals 1996 von Pinkard et al. beschrieben [1]. Die meisten Patient:innen sind asymptomatisch. Gelegentlich treten lokale Beschwerden wie Thoraxschmerzen oder Dyspnoe auf. Meist betrifft die Erkrankung junge bis mittelalte Erwachsene und tritt solitär oder multipel auf. Eine maligne Transformation oder Metastasierung ist bisher nicht bekannt. Whole-Exome-Sequenzierungen haben potenzielle molekulare Veränderungen identifiziert, diese beruhen jedoch auf kleinen Fallzahlen und haben derzeit keine diagnostische Relevanz. Die Therapie der Wahl ist eine chirurgische Resektion, die in der Regel kurativ ist, wobei lokale Rezidive beschrieben wurden. Nach aktueller WHO-Klassifikation (2021) [2] basiert die Diagnose hauptsächlich auf der Histomorphologie: eine zellarme Fibrose mit Verkalkungen. Die Immunhistochemie dient vor allem dem Ausschluss der wichtigsten Differenzialdiagnosen, zu denen aufgrund der pleuralen Lokalisation insbesondere der solitäre fibröse Tumor (SFT), die Desmoidfibromatose und der inflammatorische myofibroblastäre Tumor (IMT) gehören. Bei Beteiligung des Mediastinums ist auch an eine sklerosierende Mediastinitis zu denken. Histologisch kann das Bild auch eine Amyloidose oder kalzifizierte Pleuraplaques imitieren
